# Differential interleukin-6/Stat3 signaling as a function of cellular context mediates Ras-induced transformation

**DOI:** 10.1186/bcr2725

**Published:** 2010-10-07

**Authors:** Kenneth Leslie, Sizhi P Gao, Marjan Berishaj, Katrina Podsypanina, Hao Ho, Lionel Ivashkiv, Jacqueline Bromberg

**Affiliations:** 1 Department of Medicine, Memorial Sloan Kettering Cancer Center, 1275 York Avenue, New York, NY 10021, USA; 2 Department of Medicine, Arthritis and Tissue Degeneration Program, Hospital for Special Surgery, 535 East 70th St., New York, NY 10021, USA; 3 Mammary Cancer Biology Research, Institut de Recherches Cliniques de Montreal (IRCM), 110, Avenue des Pins Ouest, Montreal, QC H2W 1R7, Canada

## Abstract

**Introduction:**

Tyrosine phosphorylated signal transducer and activator of transcription 3 (pStat3) is expressed in numerous cancers and is required for mediating tumorigenesis. Autocrine and paracrine interleukin (IL)-6 signaling is the principal mechanism by which Stat3 is persistently phosphorylated in epithelial tumors including breast, lung, colon and gastric cancer. The Ras oncogene mediates cellular transformation without evidence of pStat3 in cultured cells. However, non-tyrosine phosphorylated Stat3 was shown to function as a transcriptional activator, localize to the mitochondria and regulate ATP synthesis and mediate cell migration. Here we examined the role of Stat3 in Ras mediated transformation.

**Methods:**

Ha-rasV12 transformed mammary epithelial cells (MCF10A-Ras) cells were transduced with a Stat3shRNA, IL-6shRNA and/or treated with inhibitors of Janus kinases (JAKs) to examine the role of the IL-6 signaling pathway in Ras mediated migration, invasion and tumorigenesis.

**Results:**

Cellular migration, invasion, anchorage independent growth and tumorigenesis were largely abrogated in the Stat3-reduced cells compared to control cells. Analysis of MCF10A-Ras tumors revealed high levels of pStat3 and interleukin-6. Tumors derived from transgenic MMTV-K-Ras mice were also found to express pStat3 and IL-6. MCF10A-Ras cells, when grown in a three-dimensional Matrigel culture system revealed the appearance of the junctional protein E-Cadherin as a consequence of reducing Stat3 levels or inhibiting Stat3 activity. Decreasing IL-6 levels in the MCF10A-Ras cells abrogated tumorigenesis and reduced cell migration. By isolating Ras-expressing primary tumors and serially passaging these cells in two-dimensional culture led to a decrease in IL-6 and pStat3 levels with the reappearance of E-Cadherin.

**Conclusions:**

The cellular and environmental context can lead to differential IL-6/pStat3 signaling and a dependency on this cytokine and transcription factor for migration, invasion and tumorigenesis.

## Introduction

The Signal transducers and activators of transcription (Stat) family of proteins are transcription factors known for their role as integrators of cytokine and growth factor receptor signaling and are required for cell growth, survival, differentiation, and motility [1,2]. Stat activation is dependent upon tyrosine phosphorylation, which induces dimerization via reciprocal phosphotyrosine-src homology domain 2 (phosphotyrosine-SH2) interaction between two Stat molecules. Activated Stat's translocate to the nucleus where they bind to consensus promoter sequences of target genes and activate their transcription [3]. In normal cells, Stat tyrosine phosphorylation is transient. However, in numerous cancer-derived cell lines and in an ever growing number of primary tumors, Stat proteins (in particular Stat3) are persistently tyrosine phosphorylated [4]. Stat3 is found to be constitutively phosphorylated to high levels in >50% of breast cancer derived cell lines and in >30% of breast adenocarcinomas and may be a poor prognostic indicator [5,6]. Constitutive activation of Stat3 in epithelial cancers and cancer derived cell lines is frequently due to aberrant autocrine or paracrine IL-6 signaling [7]. Inhibition of Stat3 activity in tumor-derived cell lines both *in vitro *and *in vivo*, by the introduction of antisense, small interfering RNA, decoy molecules, dominant-negative Stat3 constructs, and/or blockade of tyrosine kinases has been associated with growth arrest, apoptosis, decreased angiogenesis and invasion [2,4,8,9]. More recently, non-canonical functions for Stat3 have been identified including non-tyrosine phosphorylated Stat3 mediating transcriptional activation, non-tyrosine phosphorylated Stat3 binding to stathmin a microtubule associated protein and regulating migration, non-tyrosine phosphorylated Stat3 regulating metabolic functions in the mitochondria leading to Ras-dependent transformation [10-12].

The ras proto-oncogene encodes a guanine nucleotide binding protein that plays an essential role in diverse cellular responses, including cell proliferation and differentiation [13]. Although ras mutations are infrequent in human breast cancers, elevated amounts of the ras protein have been found in 60 to 70% of human primary breast carcinomas [14]. Ras expression has been suggested to be a marker of tumor aggressiveness in breast cancer, including the degree of invasion into fat tissue, infiltration into lymphatic vessels and tumor recurrence [14-16]. Rodent fibroblasts and human mammary epithelial cell lines transformed by the H-Ras oncogene do not express tyrosine phosphorylated Stat3 [17-19]. Moreover, non-tyrosine phosphorylated Stat3 was demonstrated to regulate metabolic functions in the mitochondria leading to Ras-dependent transformation [20].

Here we further investigated the role of non-tyrosine phosphorylated Stat3 in Ras-mediated mammary tumorigenesis. Specifically, we examined the consequences of reducing Stat3 levels in Ras transformed mammary epithelial cells. We determined that Stat3 deficient Ras transformed MCF10A cells were less capable of mediating migration, invasion and tumorigenesis than the control MCF10A-Ras cells. Surprisingly, tumors derived from MCF10A-Ras cells expressed high levels of tyrosine phosphorylated Stat 3 (pStat3) as did mammary tumors from MMTV-expressing K-Ras mice. Furthermore, the interleukin-6 ligand (IL-6) which was recently shown to be a principal regulator of Stat3 activation in breast cancer [6], was found to be elevated in both MCF10A-Ras and MMTV-K-Ras tumors. In addition, growth of MCF10A-Ras cells in the presence of basement membrane proteins (Matrigel) resulted in high levels of pStat3. Reduction of Stat3 levels or inhibition of its activity led to the up-regulation of E-cadherin in MCF10A-Ras cells. We demonstrated that culturing and passaging primary Ras-expressing tumors from 3-D to 2-D resulted in a diminution of pStat3 and IL-6 levels suggesting that depending on the context in which MCF10A-Ras expressing cells are grown can significantly alter the levels of pStat3 and the subsequent behavior of the cells.

## Materials and methods

### Plasmids, protein extraction, Western blot analysis, EMSA and RNA analysis

The pBabe-H-RasV12 construct was a gift from P. Sicinski (Dana-Farber Cancer Institute) [21]. Stat3shRNA lentiviral and scrambled control shRNA constructs were previously described [22]. The pSuper-Il-6 shRNA-GFP retroviral construct was generated by substituting PKG-puro with CMV-GFP. pSuper IL-6 shRNA was a gift from C. Counter [23]. Nuclear and cytoplasmic extracts were prepared as previously described [24]. Radioimmunoprecipitation assay (RIPA) buffer extracts were utilized in the protein extraction of all tissues and Western blots were carried out as previously described [25]. Protein concentrations were determined using the Bradford assay (BioRad, Hercules, CA, USA). EMSAs were carried out as previously described [26] by using a radiolabeled high-affinity m67 DNA binding probe and an anti-Stat3 antibody for supershifting (Stat3 (K-15): sc-483 X, Santa Cruz Biotechnology, Santa Cruz, CA, USA). RNA was isolated using the RNeasy kit (QIAGEN, Valencia, CA, USA). Two micrograms of total RNA was used for IL-6 and β-actin RT-PCR using an iScript RT-PCR kit (Bio-Rad) according to the manufacturer's instructions. Sequences of primers for amplification of the IL-6 gene were as follows: forward primer, *5'-TAGCCGCCCCACACAGACAG-3*'; reverse primer, *5'-GGCTGGCATTTGTGGTTGGG-3*'. The β-actin primers were as follows: forward primer, *5'-CGTGCGTGACATTAAGGAGA-3*'; reverse primer, *5'-TGATCCACATCTGCTGGAAG-3*'. For quantitative PCR 1 ug of total RNA was reverse-transcribed using the Thermoscript RT-PCR system (Invitrogen) at 52°C for one hour. 20 ng of resultant cDNA was used in a Q-PCR reaction using an iCycler (Biorad) and pre-designed TaqMan ABI Gene expression Assays (Hs00985639_m1 for IL-6). Amplification was carried for 40 cycles (95°C for 15 seconds, 60°C for 1 minute). To calculate the efficiency of the PCR reaction, and to assess the sensitivity of the assay, we also performed a seven-point standard curve (5, 1.7, 0.56, 0.19, 0.062, 0.021, and 0.0069 ng). To obtain normalized qPCR values for IL-6, triplicate cycle threshold values were averaged, amounts of target were interpolated from the standard curves and normalized to HPRT (hypoxanthine guanine phosphoribosyl transferase, assay Hs99999909_m1). Antibodies used were: anti-tubulin monoclonal antibody (1:2500) (Sigma, St. Louis, MO, USA); Anti-H-Ras polyclonal antibody (1:1000) (Santa Cruz Biotechnology); Anti-Stat 3 and anti-Tyr 705 Stat3 polyclonal antibodies (1:1000) (Cell Signaling, Danvers, MA, USA) and Anti-E-cadherin monoclonal antibody (1:1000) (Zymed Laboratories, San Francisco, CA, USA).

### Cell culture, growth curves, retroviral/lentiviral infections

MCF10A and 293T cells were obtained from the American Type Culture Collection. MCF10A cells were cultured as previously described [27]. 293T cells were grown in Dulbecco's modified Eagle medium (DMEM) containing 10% Cosmic Calf serum (Hyclone, Logan, UT, USA). Cells were plated on plastic coated Matrigel, Fibronectin, Laminin, CollagenI, CollagenV (BD Biosciences). Human IL-6 was used at 10 ng/ml (R&D Systems, Minneapolis, MN, USA). To measure cell growth, 2 × 10^4 ^cells per well (2 × 10^3^/cm^2^) were plated into six-well dishes. Cells were counted at Days 1, 2, 3, 4, 5 and 6. Each data point represents the mean value from triplicate wells. All transfections were carried out using Superfect (QIAGEN). Retroviral infections were carried out using the RetroMax Retroviral Expression System pCL-Ampho (Imgenex, San Diego, CA, USA) according to the supplier. Clonal selection was carried out using puromycin (2 ug/ml) (Sigma). Lentiviral infections were performed using lentivirus-based vectors encoding shRNA IRES eGFP targeted to either Stat3 or IL-6 transcripts, and were generated by transient co-transfection of 293T cells with a three-plasmid combination, as described previously [28]. EGFP expressing cell populations positive for each shRNA were sorted by FACS.

### Migration/invasion

Migration/invasion experiments were performed as described previously [29,30]. Briefly, cells (5 × 10^4^) were starved for 16 hours in media lacking EGF and were subsequently applied to an 8-μm pore cell culture inserts or in Matrigel Invasion Chambers (Falcon/BD Labware, Franklin Lakes, NJ, USA). MCF10A media containing 2% horse serum and EGF was used as the chemoattractant with or without IL-6 (10 ng/ml). After 16 h of incubation, the cells were stained with crystal violet and counted. Each condition was assayed in triplicate, experiments were performed independently at least three times, and the results were expressed as the number of cells per field.

### Morphogenesis assay

Morphogenesis assays were carried out as previously described [27]. Growth Factor Reduced Matrigel was obtained from BD Biosciences (BD No.354230). For inhibitor incubation, the following concentrations of inhibitor or antibody were used: 1 μM P6 (Calbiochem, San Diego, CA, USA), 10 μg/mL BR3 (Diaclone, Stamford, CT, USA) and 2 μg/mL anti-IL-6 522 (Cell Sciences, Conton, MA, USA). Antibodies for staining of acini were as follows: anti-phospho Stat3 # 9135, 1:50 (Cell Signaling); anti-E-Cadherin 1:200 (Zymed Laboratories). Examination of the staining of cells was carried out using a Leica inverted confocal microscope.

### Soft agar assay/tumor growth

Anchorage-independent growth in triplicate was assessed as previously described [17]. Cells (4 × 104) in 4 ml of a 0.35% agar-MCF10A media solution were plated in triplicate on 35-mm-diameter dishes containing a 0.7% agar plug (BiTek, DIFCO Laboratories, Detroit, MI, USA). Colonies were stained with 3-(4,5-dimethylthiazol-2-yl)-2,5-diphenyltetrazolium bromide (Sigma) and counted after three weeks. Cells (107) were harvested and mixed with an equal volume of Matrigel (Becton Dickinson Labware, Bedford, MA, USA), and 200-μl doses were injected into the flanks of six- to eight-week-old male NCr athymic nude mice (NCI, Frederick, MD, USA). Tumor sizes were measured after four weeks and tumor volume was calculated using the formula length × width × depth × 1/2. Each cell line was injected in a minimum of five animals.

### Isolation of extracts and cell lines from tumors: MMTV-KRas tumors and MCF10A Ras

Mammary tumors from MMTV-KRas mice and xenograft MCF10A-Ras tumors were dissected [31]. Half of the tumors were frozen in liquid nitrogen, and ground to a powder for RNA and protein analysis. The remaining tumors were mechanically dissociated with scalpels and enzymatically digested in a) DMEM: F-12 with 1 mM glutamine, 5 μg/ml insulin, 500 ng/ml hydrocortisone, 10 ng/ml epidermal growth factor, 20 ng/ml cholera toxin, 5% horse serum, 300 U/ml collagenase and 100 U/ml hyaluronidase (Sigma) for one hour at 370C followed by 0.1 mg/mL DNase treatment (Worthington, Lakewood, NJ, USA) for one minute subsequently washed in DMEM with 10% FBS and resuspended and plated in MEGM media (Cambrex, Walkersville, MD, USA) (for MMTV-KRas tumors) b) 0.25% trypsin for 5' at 37°C followed by washing in DMEM with 10% FBS and plated in MEGM media (for MCF10A Ras tumors). Cells were plated and subcultured daily. Supernatants were saved for IL-6 ELISA's, and RIPA extracts were made for protein analysis. ELISA analysis for human and murine IL-6 was performed using the manufacturer's instructions (Cell Sciences).

### Immunofluoresence and Immunohistochemistry

Tumors were fixed in 4% paraformaldehyde and embedded in paraffin. Tissue sections were stained for IL-6 using anti-IL-6 (1:200; Abcam, Cambridge, MA, USA) using previously described methods [32]. Imaging was carried out on a Leica Inverted Confocal Microscope.

### Statistical analysis

Data are expressed as means ± standard deviation (SD). The statistical significance of differences was evaluated using an unpaired, non-parametric Student's *t*-test. Significant differences between experimental groups were * *P *< 0.05 or ** *P *< 0.01.

## Results

### Stat3 is required for Ras-mediated migration, invasion and tumorigenesis of human mammary epithelial cells

**Figure 1:**
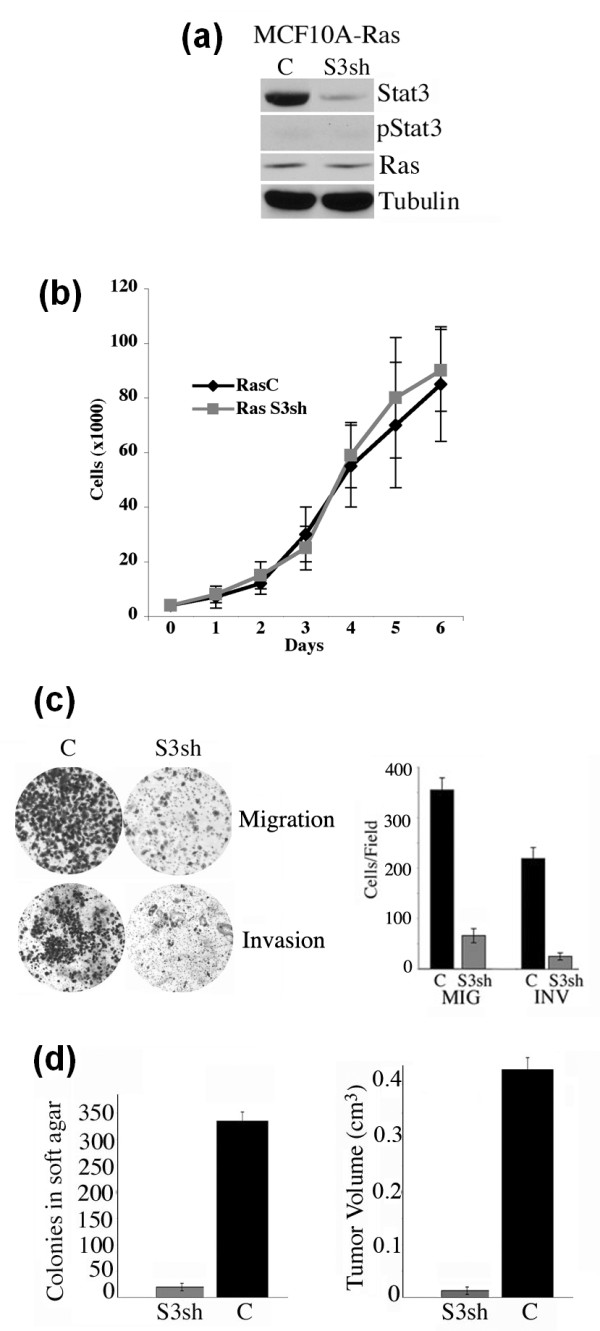
**Stat3 is required for the migratory, invasive and tumorigenic phenotype of Ras transformed MCF10A cells**. **(a) **Extracts from Ras transformed MCF10A cells (MCF10A-Ras) expressing control (C) or Stat3 shRNA (S3sh) were analyzed for levels of Stat3, tyrosine phosphorylated (pStat3), Ras and Tubulin by Western blot analysis. **(b) **MCF10A-Ras cells expressing control (C) or Stat3 shRNA (S3sh) were plated in six-well dishes and cell numbers were determined daily for seven days. Each data point represents the mean value from triplicate wells. **(c) **MCF10A-Ras cells expressing control (C) or Stat3 shRNA (S3sh) were plated into Boyden chambers and cell migration (MIG) and invasion (INV) was determined with crystal violet staining after 24 hrs. Results are expressed as cells/field (mean ± SD of triplicates from three independent experiments). **(d) **Colony formation in soft agar of MCF10A-Ras cells expressing control (C) or Stat3 shRNA (S3sh) (mean ± SD of triplicates from three independent experiments). Tumor growth in nude mice using MCF10A-Ras cells expressing control (C) or Stat3 shRNA (S3sh) was determined (mean ± SD from four independent injections).

We examined the role of Stat3 in H-RasV12 mediated cell migration, invasion and cellular transformation using H-RasV12 transformed mammary epithelial cells (MCF10A-Ras). MCF10A cells are a spontaneously immortalized human breast epithelial cell line, mutant in the cdk inhibitor p16, yet has many of the characteristics of normal breast epithelium, do not form tumors in nude mice nor form colonies in soft agar, but undergo transformation upon the introduction of Ha-Ras [33-35]. The H-RasV12 oncogene was introduced into MCF10A cells by retroviral gene transfer, and Ras expressing cells were selected in puromycin containing media. A Stat3sh or scrambled control shRNA-GFP constructs were introduced into H-RasV12 transformed MCF10A cells by lentiviral infection and sorted for GFP expression (Stat3sh and control lines respectively). Tyrosine phosphorylated Stat3 was undetectable in the MCF10A-Ras cells (Figure 1a). Stat3sh expressing cells displayed lower levels of total Stat3 protein by Western blot analysis and Ras protein levels were constant (Figure 1a). The morphology and growth rates of the Stat3sh cells were similar to control cells, as were their requirements for defined media components including EGF (Figure 1b and data not shown).

Ras transformed cells have increased invasive and migratory potential over control non-transformed cells [36]. We investigated whether Stat3 may be required for this phenotype and compared Stat3sh to control cells (Figure 1c). The Stat3sh expressing cells displayed an approximately four-fold decrease in migration in a Boyden chamber assay, compared to control cells (Figure 1c). Additionally, we observed a nearly 10-fold reduction in invasion through a matrigel coated insert in the Stat3sh cells compared with controls. Hence, Stat3 is a modulator of the invasive and migratory potential of Ras transformed mammary epithelial cells.

The finding that Stat3 is required for invasion and migration in Ras transformed MCF10A cells led us to determine if Stat3 might also be required for tumorigenesis. Anchorage-independent-growth is a measure of a cells capacity to grow in three dimensions, without contacting a basement membrane. We next determined whether Stat3 expression affects anchorage independent growth of MCF10A-Ras cells. Control cells displayed robust colony formation while Stat3sh cells formed very few colonies (approximately 10-fold less) (Figure 1d). Tumorigenesis was determined by injecting both control and Stat3sh cells into nude (nu/nu athymic) mice. Mice injected with Stat3sh cells formed tiny acellular tumors (0.007 cm3) relative to control cells (0.412 cm3) (Figure 1d). Taken together, these results indicate that Stat3 is required for Ras mediated transformation of MCF10A cells.

### Ras expressing mammary tumors exhibit high levels of activated Stat3 and IL-6

**Figure 2:**
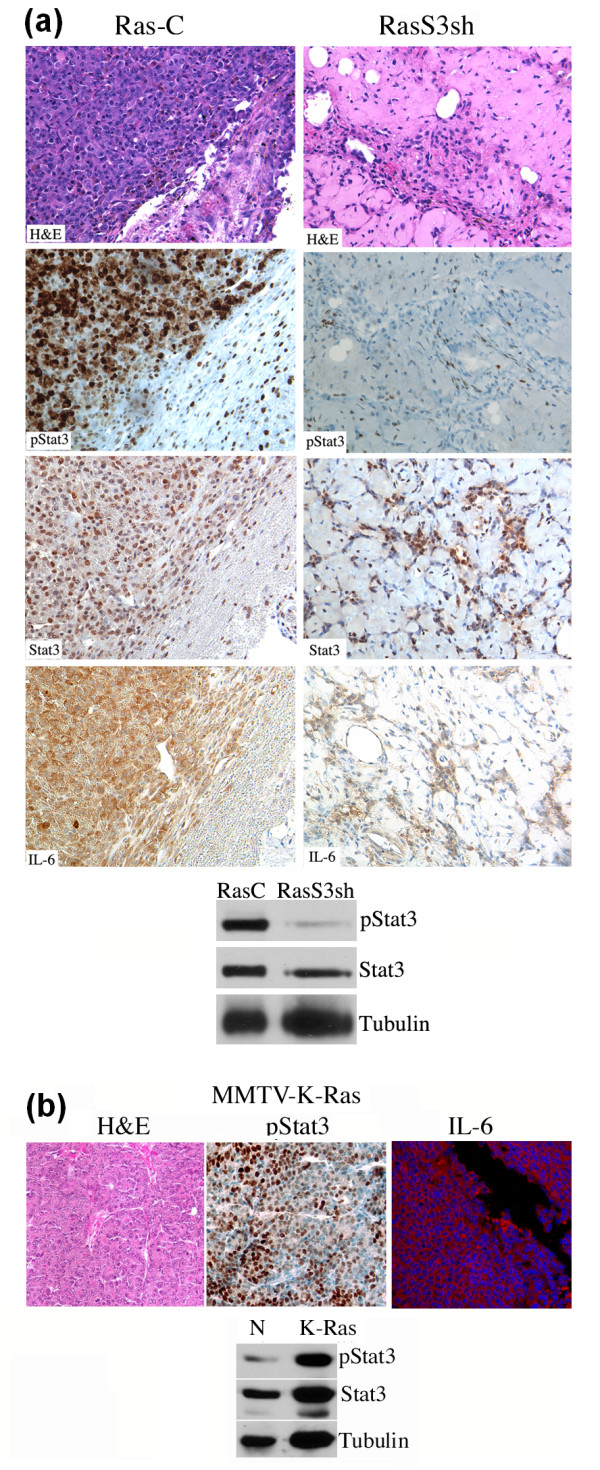
**Ras expressing mammary tumors exhibit high levels of activated Stat3 and IL-6**. **(a) **Paraffin embedded tumors from MCF10A-Ras cells expressing control (Ras-C) or Stat3shRNA (RasS3sh) were examined by hematoxyline and eosin (H&E), pStat3 and IL-6 by immunohistochemistry. Extracts from MCF10A-Ras expressing control (C) or Stat3 shRNA (S3sh) tumors were analyzed for pStat3 and Tubulin by Western blot analysis. **(b) **Paraffin embedded tumors from MMTV-promoter driven K-Ras (MMTV-K-Ras) were analyzed by hematoxyline and eosin stain (H&E), for pStat3 by immunohistochemistry and for IL-6 by immunofluorescence. Extracts from normal mammary tissue (N) or MMTV-K-Ras (K-Ras) were analyzed for pStat3, Stat3 and Tubulin.

We next examined the levels of pStat3 in these tumors by immunohistochemical and Western blot analyses. Surprisingly, we observed high pStat3 levels in the control tumors (both tumor cells and adjacent stromal cells), while the Stat3sh tumors had very low levels of pStat3 (Figure 2a). The cells expressing pStat3 and total Stat3 within the Stat3sh tumors were principally non-tumor cells (Figure 2a). We recently showed that a principal mechanism of Stat3 activation in breast and lung cancers is through autocrine production of IL-6 [6,32]. Furthermore, it was shown that a number of Ras transformed cells express high levels of IL-6 which promotes angiogenesis and tumorigenesis [23]. We therefore analyzed these tumor samples for IL-6 expression by immunohistochemistry and determined that control tumors expressed high levels of IL-6 (Figure 2a). To demonstrate that our observations were not specific to the MCF10A-Ras cell line, we examined mice expressing the K-Ras oncogene within the mammary gland (MMTV-K-Ras). These tumors also expressed high levels of pStat3 (by immunohistochemistry and Western blot analysis) and IL-6 (by immunofluoresence) (Figure 2b).

### Paracrine IL-6 enhances autocrine IL-6/pStat3 signaling and migration in MCF-10A-Ras cells

**Figure 3:**
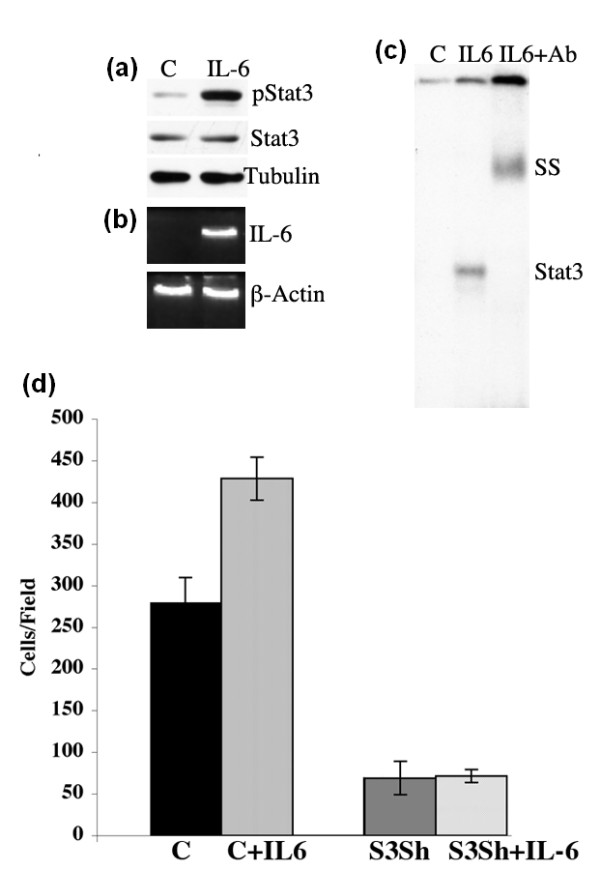
**Exogenous IL-6 enhances pStat3 levels, binding activity and cell migration in MCF10A-Ras cells**. **(a) **Extracts from MCF10A-Ras cells (C) and MCF10A-Ras treated with IL-6 (10ng/ml) for one hour (IL-6) were analyzed by Western blot analysis for pStat3, Stat3 and Tubulin **(b) **Human *IL-6 *mRNA levels from MCF10A-Ras cells (C) and MCF10A-Ras cells treated with IL-6 for four hours (IL-6) were determined by RT-PCR and normalized to β-Actin. **(c) **EMSA was performed with nuclear extracts from the cell lines described in a. Stat3 DNA binding was supershifted with anti-Stat3 antibody (IL6+Ab). **(d) **MCF10A-Ras cells (C) or Stat3 shRNA (S3sh) were plated into Boyden chambers and cell migration was determined in the absence or presence of IL-6 (C+IL6, S3Sh+IL-6) with crystal violet staining after 24 hrs. Results are expressed as cells/field (mean ± SD of triplicates from three independent experiments).

It was recently determined that exogenous sources of IL-6 could enhance autocrine production of IL-6 in models of breast cancer where IL-6/pStat3 levels were very low [37]. Furthermore, IL-6 has been shown to promote an epithelial-mesenchymal transition in breast cancer which correlated with enhanced invasion [38]. Given our observation that MCF10A-Ras cells express IL-6 and pStat3 in 3-D, we wished to determine whether exogenous IL-6 could lead to Stat3 phosphoryaltion and inducible expression of endogenous IL-6 in MCF10A-Ras cells grown in 2-D. We treated cells with IL-6 and observed a marked increase in pStat3 levels by Western blot analysis, Stat3 DNA binding activity by electrophorectic mobility shift assay (EMSA) as well as IL-6 mRNA levels (Figure 3a, b, c). We also examined the effect of exogenous IL-6 on MCF10A-Ras cell migration and determined that IL-6 enhanced MCF10A-Ras cell migration in a Stat3 dependent manner as IL-6 could not promote migration in RasS3Sh cells (Figure 3d). Thus, paracrine or exogenous sources of IL-6 enhances pStat3 levels, Stat3 binding activity and cell migration in a Stat3 dependent manner.

### MCF10A-Ras cells grown in Matrigel express IL-6 and pStat3 which regulate E-Cadherin levels

**Figure 4:**
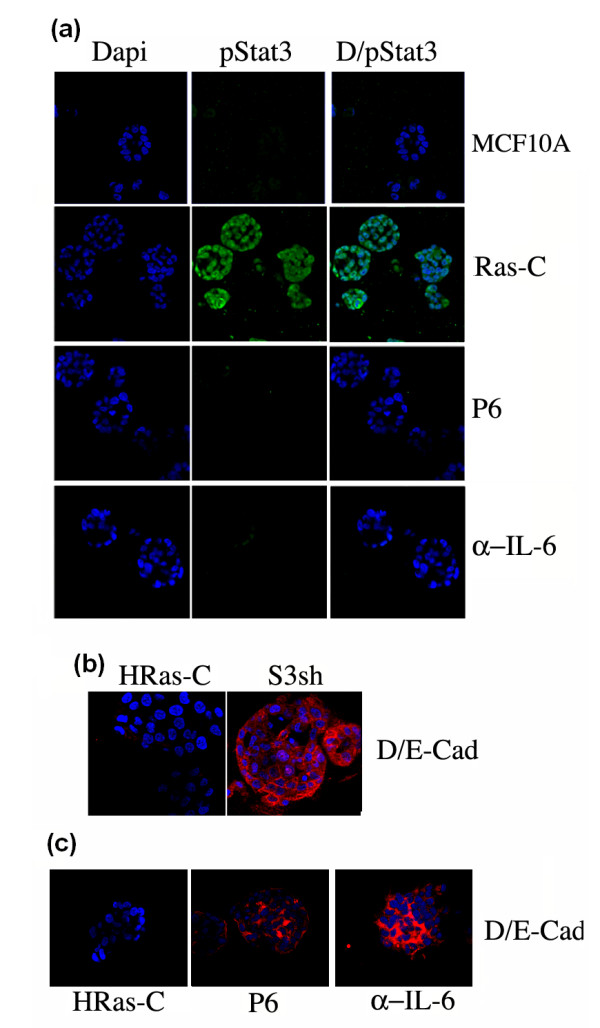
**3-D morphogenic assays of MCF10A-Ras cells display IL-6/Jak/pStat3 signaling which regulate E-Cadherin levels**. **(a) **MCF10A cells were grown in Matrigel as were MCF10A-Ras cells which were stained for pStat3 and Dapi by immunofluorescence treated with DMSO control (Ras-C), P6 (a pan-Jak inhibitor) or a blocking antibody to IL-6 (α-IL-6). **(b) **MCF10A-Ras cells expressing control (HRas-C) or Stat3 shRNA (S3sh) were grown in Matrigel and stained with Dapi and E-Cadherin (D/E-Cad) by immunofluorescence. **(c) **MCF10A-Ras cells were grown in matrigel and treated with DMSO (HRas-C), P6 or a blocking antibody to IL-6 (α-IL-6) and stained for Dapi and E-Cadherin (D/E-Cad).

To further characterize the requirement for Stat3 in MCF10A-Ras cells, we utilized a Matrigel assay to examine growth in three-dimensions (3-D). The culture of MCF10A mammary epithelial cells on a defined basement membrane (Matrigel) results in the formation of polarized, hollow acini which recapitulates several aspects of glandular architecture *in vivo *[27]. Furthermore, oncogenes introduced into MCF10A cells disrupt this ordered process and elicit distinct morphological phenotypes [27]. MCF10A-Ras cells grew as amorphous structures which were not hollow and expressed high levels of pStat3 as determined by immunofluorescence (Figure 4a). MCF10A cells were also plated in matrigel revealing hollow acini which were negative for pStat3 by immunocytochemistry (Figure 4a). Inhibition of IL-6 signaling using an anti-IL6 blocking antibody or using a pan-Jak inhibitor (P6) led to a reduction in pStat3 levels (Figure 4a). Matrigel is a mixture of extracellular matrix (ECM) proteins composed primarily of laminin and collagen. We tested the role of matrigel and its components for the ability to enhance Stat3 phosphorylation and determined that matrigel and laminin were capable of inducing pStat3 (Supplemental figure S1 in Additional file 1). Thus, the growth of MCF10A-Ras on defined ECM proteins can enhance Stat3 activation. A role for Stat3 in MCF10A cell growth and acini formation was also examined. A reduction in Stat3 had no effect on the morphology of the acini nor on growth in 2-D (Supplemental figure S2 in Additional file 2). MCF10A-Ras cells lack E-cadherin expression which marks organized cell-cell contacts. Stat3sh cells continued to grow as filled acini but interestingly E-Cadherin expression was restored (Figure 4b). Similarly, treatment of MCF10A-Ras cells with inhibitors of IL-6/Jak/pStat3 signaling led to the expression of E-cadherin. Treatment of MCF10A-Ras 3-D structures with a pan-Jak inhibitor did not lead to a hollowing out of the structures despite the reappearance of E-Cadherin (Supplemental figure S3 in Additional file 3). IL-6/Stat3 signaling has been shown to inhibit E-Cadherin expression in models of prostate and breast cancer [38,39]. This data suggest that phosphorylated Stat3 has a role in regulating Cadherin expression and the loss of a requirement for cell-cell contacts in transformed cells.

### IL-6 is required for tumorigenesis of Ras transformed MCF10A cells

**Figure 5:**
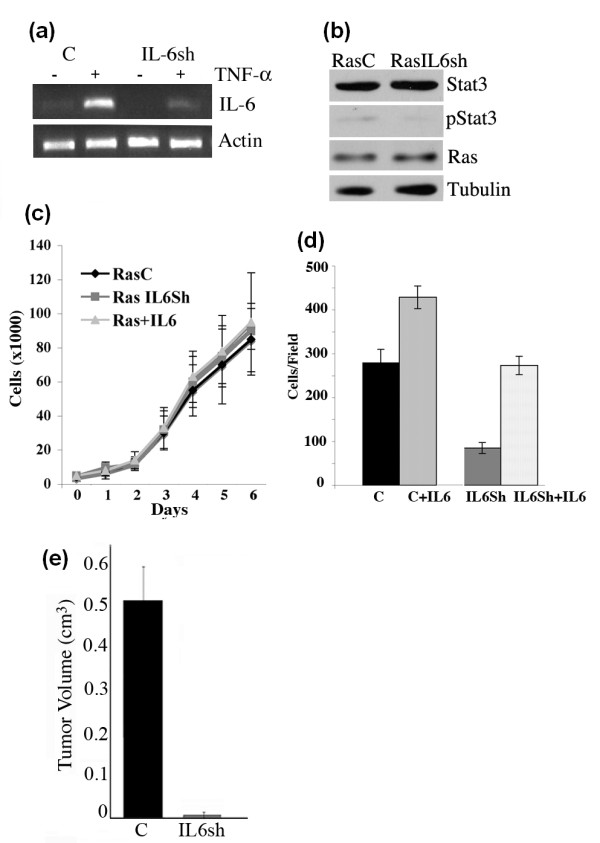
**IL-6 is required for tumorigenesis of Ras transformed MCF10A cells**. **(a) **Ras transformed MCF10A cells expressing control (C) or IL-6 shRNA (IL-6sh) were stimulated with TNF-α and analyzed for levels of IL-6 and normalized to actin by RT-PCR. **(b) **Extracts from MCF10A-Ras (RasC) and MCF10AIL6Sh (RasIL6sh) were analyzed for Stat3, pStat3, Ras and Tubulin levels. **(c) **MCF10A-Ras (RasC) and MCF10AIL6Sh (RasIL6Sh) cells were plated in six-well dishes and cell numbers were determined daily for seven days. Additionally, RasC cells were treated with IL-6 (10 ng/ml) (Ras+IL6) daily and cell numbers were determined. Each data point represents the mean value from triplicate wells. **(d) **MCF10A-Ras (C) and MCF10AIL6Sh (IL6Sh) cells were plated into Boyden chambers and cell migration was determined in the absence or presence of IL-6 (C+IL6, S3Sh+IL-6) with crystal violet staining after 24 hrs. Results are expressed as cells/field (mean ± SD of triplicates from three independent experiments). **(e) **Tumor growth in nude mice using MCF10A-Ras cells expressing control (C) or IL-6 shRNA (IL-6sh) was determined (mean ± SD from five independent injections).

To investigate the relationship between cell transformation and IL-6 signaling, we introduced an shRNA construct targeting the IL-6 mRNA transcript into Ras transformed MCF10A cells. IL-6 shRNA functionality was confirmed using semi-quantitative RT-PCR analysis on cells stimulated with TNF-α, a ligand known to induce IL-6 mRNA production (Figure 5a). IL-6 transcript levels were significantly reduced in the IL-6 shRNA expressing cells. Total Stat3, pStat3 and Ras levels were unchanged by the loss of IL-6 (Figure 5b). However, the addition of exogenous IL-6 led to robust Stat3 tyrosine phosphorylation (data not shown). MCF10A-Ras cells expressing either control shRNA or the IL-6 shRNA grew at similar rates when grown on plastic (2-D) (Figure 5c). Furthermore, the chronic administration of IL-6 to MCF10A-Ras cells did not increase their proliferation in 2-D (Figure 5c). We examined the loss of IL-6 on cell migration and determined that MCF10A-Ras IL-6Sh cells (IL6Sh) did not migrate as well as control cells but the addition of IL-6 could restore cell migration (Figure 5d). Tumorgenicity was also assessed. MCF10A-Ras (C) cells were injected into the flanks of nu/nu athymic mice and tumors grew as expected (>0.4 cm3), while IL-6 shRNA expressing cells failed to form tumors, further implicating IL-6 signaling as a key pathway in Ras-mediated transformation (Figure 5e).

### The growth environment of Ras transformed MCF10A cells affects IL-6 production and the phosphorylation status of Stat3

**Figure 6:**
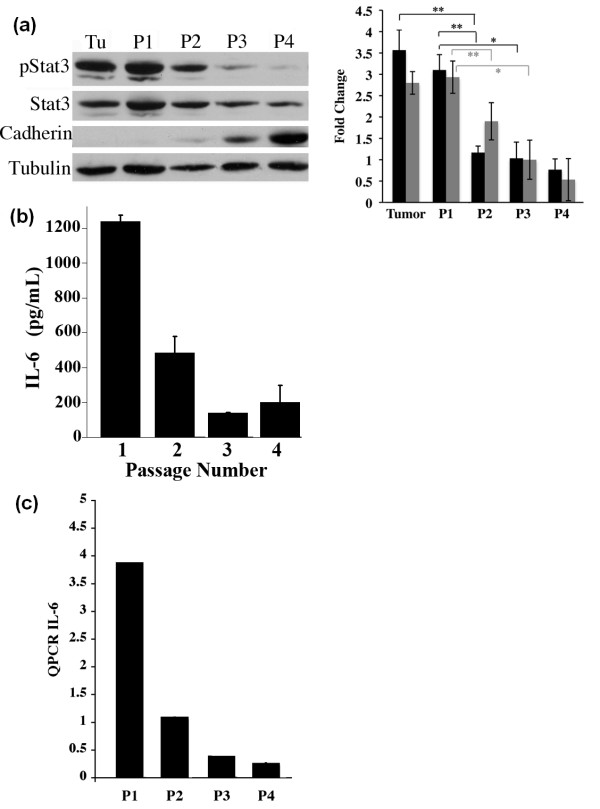
**Growth of MCF10A-Ras cells in two- versus three-dimensions alters the expression levels of pStat3, IL-6 and E-Cadherin**. **(a) **Tumors arising from immunocompromised mice injected with MCF10A-Ras cells were cultured and passaged four times on tissue culture plates. Extracts were isolated from an MCF10A-Ras primary tumor (Tu) and cultured cells following serial passage (P1, P2, P3 and P4) and analyzed for pStat3, Stat3, E-Cadherin and Tubulin by Western blot. Quantitative evaluation of pStat3/Stat3 (grey bars) and pStat3/Tubulin (black bars) immunoblots by densitometry. Results are representative of three individual experiments. Statistical significance is indicated with asterisks (*, *P *< 0.05; **, *P *< 0.01). **(b) **IL-6 levels by of supernatants from passaged cells were determined by ELISA. C. RNA from passaged cells (P1, P2, P3, P4) was analyzed for human IL-6 mRNA levels by real time RT-PCR (Q-PCR). Results shown are IL-6 expression normalized to HPRT from triplicates.

Our data indicate that MCF10A-Ras cells when grown in two dimensions (2-D) do not express detectable levels of pStat3 (Figure 1a). However, when these same cells were grown in three dimensions (3-D), either in a Matrigel assay or as tumors in nude mice, we observed high levels of pStat3 (Figures 2 and 4). We then asked whether the presence of pStat3 observed in 3-D growth was reversible upon culturing in 2-D. MCF10A-Ras tumors were surgically removed, and the epithelial cell population was serially cultured over four days. Protein extracts and RNA was isolated from the initial tumor growth, as well as from each passage of these cells. Additionally, the supernatant from the cultured cells of each passage was obtained in order to measure IL-6 levels. MCF10A-Ras tumors exhibited high levels of pStat3, however the passaging of these cells over time resulted in decreased pStat3 levels (Figure 6a). The decrease in pStat3 by Western blot correlated directly with a decrease in IL-6 protein levels in the cultured cell supernatants as determined by ELISA (Figure 6b) and of IL-6 mRNA levels by real-time PCR (Figure 6c). Conversely, we observed an increase in E-Cadherin levels as pStat3 and IL-6 levels were decreasing (Figure 6a). Similar results were obtained from MMTV-K-Ras tumors cultured as described above, whereby pStat3 and IL-6 levels were markedly decreased upon two passages (data not shown). Hence, the growth environment (growth in 2-D versus 3-D), markedly affects the IL-6/Jak/Stat3 signaling pathway in Ras transformed mammary epithelial cells.

## Discussion

We sought to determine the role of non-tyrosine phosphorylated Stat3 in tumorigenesis by examining the breast epithelial cell line MCF10A cells transformed with the H-RasV12 oncogene. Non-tyrosine phosphorylated Stat3 can function as a transcription factor in association with NF-kB driving expression of a number of genes involved in tumorigenesis including BCL2A1, Rho GAP6, MRAS, MET, RANTES and Cyclin B1 [11]. We examined and compared levels of these transcripts in MCF10A-Ras cells either expressing or lacking Stat3 and found no significant differences (data not shown). Furthermore, we performed gene expression profiling on these RNA populations and found only 10 transcripts that were potentially differentially regulated as a function of non-tyrosine phosphorylated Stat3 (data not shown). Thus in this cell line, it does not appear that non-tyrosine phosphorylated Stat3 plays a significant role in regulating transcription.

We examined cell proliferation and observed no differences as a function of Stat3 (Figure 1). Furthermore, stimulation of cells with exogenous IL-6 led to robust Stat3 phosphorylation but did not affect cell proliferation (Figures 3 and 5c). Thus, Stat3 (either phosphorylated or non-phosphorylated) has no significant impact on 2-D growth. These observations have previously been made demonstrating a marginal role for gp130, Stat3 or constitutively activated Stat3 (Stat3-C) in 2-D cell proliferation but a dominant one for *in vivo *growth [40,41]. In contrast to cell proliferation, we determined that Stat3 was required for migration and invasion (Figure 1). It was recently shown that Rac1 activation leads to enhanced IL-6 expression and gp130/Jak/Stat3 activation leading to gp130 dependent cell migration [42]. Activated Stat3 has been shown to mediate migration of cancer cells by regulating genes such as *integrin β6*, *tenascinC, twist *and *liv1 *[39,43-46]. In addition to its transcriptional activating function, phosphorylated Stat3 was shown to interact with focal adhesion kinase (FAK) and was shown to play a role in cell migration [10,47]. We hypothesize that migrating or invading Ras transformed MCF10A cells activate Rac1 which leads to increased IL-6 expression, Stat3 tyrosine phosphorylation and enhanced cell migration and invasion. This process can be enhanced by paracrine IL-6 and partially inhibited by reducing IL-6 levels (Figure 5).

IL-6 was shown to be expressed to high levels in numerous Ras-expressing cell lines including kidney, fibroblasts, human mammary epithelial cells and pancreatic cancer derived cell lines when grown in 2-D [23]. In contrast, we do not see any appreciable IL-6 mRNA or protein expression in Ras transformed MCF10A cells grown in 2-D (Figures 3b and 5a). Perhaps, expression levels of Ras influence IL-6 production which may have been lower in our cells than in those described by the Counter laboratory. In contrast to cells grown on plastic, we observed that MCF10A-Ras cells grown in 3-D either in basement membrane (Matrigel) cultures or as xenografts expressed high levels of IL-6 and pStat3 (Figures 2, 4 and Supplemental figure S1 in Additional file 1). In addition, MMTV-Ras transgenic mice also developed tumors expressing IL-6 and pStat3 (Figure 2). Thus, our data suggest that the environment in which Ras transformed cells are grown can regulate the expression levels of IL-6.

MCF10A cells are immortalized human mammary epithelial cells that undergo a program of apical-basolateral polarization, proliferation, growth arrest and apoptosis leading to acinar formation when grown in matrigel [48-50]. These 3-D cultures are felt to be a more relevant system to examine cell growth, cell adhesion and cell-ECM interactions recapitulating some of architectural changes observed *in vivo *[49]. Expression of activated H-Ras in MCF10A cells led to changes in the morphology of these structures from organized hollow acini to solid irregularly shaped structures lacking E-Cadherin expression (Figure 4) [51]. We previously demonstrated that prostate epithelial cells expressing a constitutively activated Stat3 (Stat3-C) had decreased E-Cadherin levels [39]. Furthermore, IL-6 stimulated mammary epithelial cells downregulate E-Cadherin expression [38]. Here we provide further evidence that pStat3 negatively regulates E-Cadherin expression in Ras transformed MCF-10A cells as inhibiting its activity led to an increase in E-Cadherin expression (Figures 4 and 6). Although Jak inhibition restored E-Cadherin expression in MCF10A-Ras cells we did not observe any hollowing out of the acini suggesting that E-Cadherin expression is insufficient to induce a reorganization of the acini or induce apoptosis of the centrally located cells (Figure 4 and Supplemental figure S3 in Additional file 3). Although Jak inhibition had no affect on the 2-D growth of MCF10A-Ras cells we did see a loss of viability in the acinar structures grown in 3-D over a seven-day period (data not shown). These observations further support the hypothesis that inhibition of IL-6/Jak/Stat3 signaling inhibits 3-D growth and tumorigenesis but not 2-D growth.

The mechanisms of IL-6 transcriptional regulation in transformed cells involves the activation and recruitment to the IL-6 promoter of a number of transcription factors including AP-1, NF-kB, (NF-IL6) or C/EBPβ and CREB [52,53]. Furthermore, a myriad of other transcription factors in association with the above mentioned proteins can modulate expression of the IL-6 gene for example nuclear hormone receptors (GR and ER), PPARγ and Stat3 [12,54-60]. IL-6 mRNA stability is also tightly regulated through the association of RNA-binding proteins (AUF1) with the 3'UTR and activation of p38 [61,62]. We examined levels of activated NF-kB, AP-1, CREB and C/EBPβ in MCF10A-Ras cells (grown in 2-D culture) by EMSA and did not observe any binding of these factors suggesting that Ras expression in MCF10A cells is insufficient to mediate activation of the IL-6 gene (data not shown). Furthermore, our data suggest that in order for Ras transformed cells to produce high levels of IL-6, cells need to be exposed to extracellular matrix proteins such as those found in matrigel (principally laminin) or to an *in vivo *environment which exposes epithelial cells to extracellular matrix proteins but also fibroblasts, endothelial cells and macrophages which produce growth factors capable of mediating IL-6 expression. When MCF10A-Ras cells were cultured on plates coated with matrigel, collagen I, collagen IV, fibronectin and laminin we found that matrigel and laminin could induce modest expression of pStat3 (Supplemental figure S1 in Additional file 1). We suggest that integrin engagement of ECM proteins can enhance pStat3 through upregulation of IL-6. Indeed, there are examples whereby integrin engagement with extracellular matrix proteins such as collagen and laminin leads to increased IL-6 production [63-65]. The work by the Bissell laboratory has demonstrated that the nature of the ECM matrix (solid or gel-like) can profoundly influence cell morphology and gene expression [66]. Furthermore, stromal cells surrounding epithelial cells secrete IL-6 and in a paracrine manner can induce epithelial cells to produce IL-6 in an autocrine manner [37,67]. Since murine IL-6 does not engage the human IL-6R, other factors are therefore implicated in promoting human IL-6 expression and Stat3 phosphorylation in these tumors [68]. For example, aberrant EGFR signaling in glioblastoma, lung cancer and MCF10A cells led to enhanced IL-6 production and signaling [32,69].

In order to determine whether the 3-D environment is required for sustained IL-6 expression by epithelial cells we cultured tumor cells (either from MCF10A-Ras xenografts or MMTV-Ras tumors) and passaged them on plastic dishes (2-D environment). After a few passages in culture, we have an enriched epithelial cell population (devoid of fibroblasts, endothelial cells and immune cells) which no longer expresses IL-6 nor pStat3 (Figure 6). These data suggest that the micro-environment (including stromal cells, endothelial cells and immune cells) is critical for the expression of the IL-6 ligand which results in activation of Stat3. A requirement for IL-6 signaling in tumor formation has been demonstrated by using IL-6 knock-down approaches as well as blocking antibodies to IL-6 in a variety of cell types [23,32,70,71]. Here we also demonstrate a requirement for IL-6 in MCF10A-Ras mediated tumor formation with no apparent effect on 2-D growth.

## Conclusions

There is increasing evidence that tumorigenesis and metastatic progression is dependent on the interactions between tumor cells and the context in which they are grown: the tumor microenvironment. Our data demonstrate cross-talk between the Ras oncogene and the IL-6/Stat3 signaling pathway through up-regulation of IL-6 as a function of the cellular context.

## Abbreviations

DMEM: Dulbecco's modified Eagle medium; ECM: extracellular matrix; FACS: flow activated cell sorting; FAK: focal adhesion kinase; GFP: green fluorescence protein; IL-6: interleukin-6; JAKs: Janus kinases; MMTV: mouse mammary tumor virus; pStat3: tyrosine phosphorylated signal transducer and activator of transcription 3; RIPA: radioimmunoprecipitation assay; shRNA: short hairpin RNA; Stat3: signal transducer and activator of transcription.

## Competing interests

The authors declare that they have no competing interests.

## Authors' contributions

KL performed and designed most of the experiments and helped write the manuscript. SG and MB performed several experiments. HH worked in the laboratory of LI generating the lentiviral Stat3shRNA. KP provided the MMTV-K-Ras mice and tumors and technical assistance. JB conceived and designed the experiments and critically revised the manuscript. All authors read and approved the final manuscript.

## Supplementary Material

Additional file 1 **Supplemental figure S1. ECM induces Stat3 phosphorylation**. Extracts from MCF10A-Ras cells plated on plastic (C)), plastic coated Matrigel (M), plastic (C), Collagen I (CI), Collagen V(CV), Fibronectin (F) and Laminin (LA) for 16 hours and were analyzed for pStat3 and Tubulin levels. Click here for file

Additional file 2 **Supplemental figure S2**. **Stat3 has no effect on acinar formation or cell growth in MCF10A cells.A**. Extracts from MCF10A cells expressing control (C) or Stat3 shRNA (S3sh) were analyzed for levels of Stat3, tyrosine phosphorylated (pStat3) and Tubulin by Western blot analysis. **B**. MCF10A cells (Control) or MCF10A Stat3Sh (S3Sh) were grown on Matrigel and form hollowed structures which were stained for pStat3 (green) and Dapi (blue) by immunofluorescence. **C**. MCF10A cells expressing control (C) or Stat3 shRNA (S3sh) were plated in six-well dishes and cell numbers were determined daily for seven days. Each data point represents the mean value from triplicate wells. Click here for file

Additional file 3 **Supplemental figure S3**. **Jak inhibition does not alter the morphology of MCF10A-Ras cells in Matrigel.** MCF10A-Ras cells were grown on Matrigel and structures were stained for Dapi by immunofluorescence treated with DMSO control (Ras-C) or P6 (a pan-Jak inhibitor) for one week. Click here for file
