# Supplementary figures and images for: Differential interleukin-6/Stat3 signaling as a function of cellular context mediates Ras-induced transformation
Source: Breast Cancer Res. 2010 Oct 7;12(5):R80. doi: 10.1186/bcr2725 (PMC3096973; doi:10.1186/bcr2725)

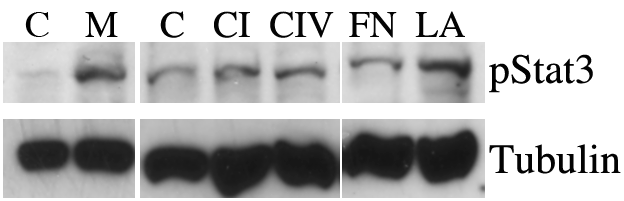

Supplement: Additional file 1 — Supplemental figure S1. ECM induces Stat3 phosphorylation. Extracts from MCF10A-Ras cells plated on plastic (C)), plastic coated Matrigel (M), plastic (C), Collagen I (CI), Collagen V(CV), Fibronectin (F) and Laminin (LA) for 16 hours and were analyzed for pStat3 and Tubulin levels. [file bcr2725-S1.TIFF]

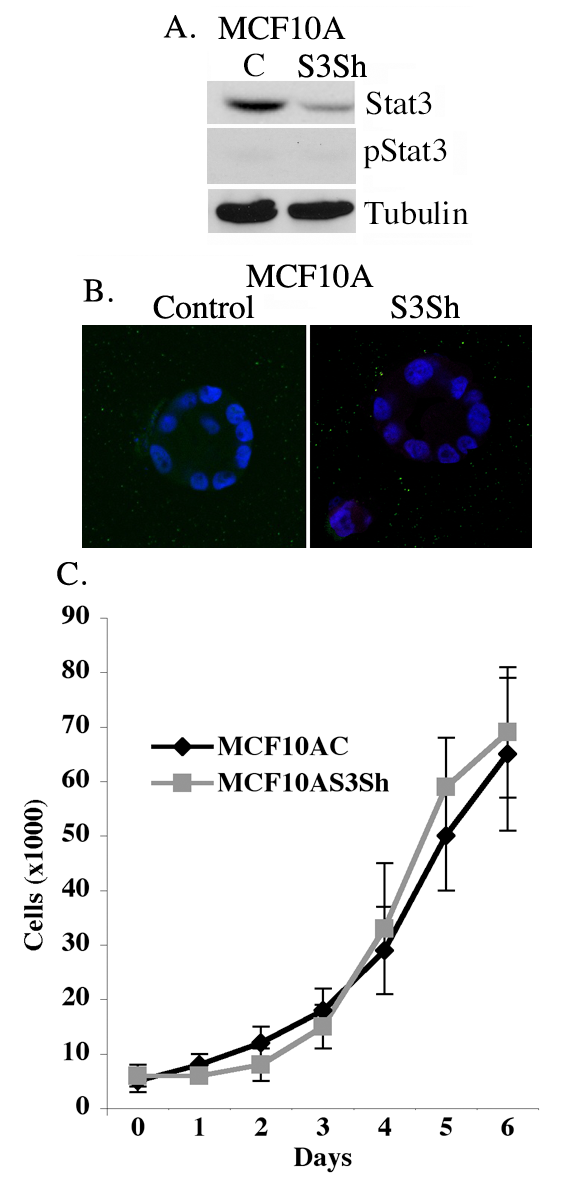

Supplement: Additional file 2 — Supplemental figure S2. Stat3 has no effect on acinar formation or cell growth in MCF10A cells.A. Extracts from MCF10A cells expressing control (C) or Stat3 shRNA (S3sh) were analyzed for levels of Stat3, tyrosine phosphorylated (pStat3) and Tubulin by Western blot analysis. B. MCF10A cells (Control) or MCF10A Stat3Sh (S3Sh) were grown on Matrigel and form hollowed structures which were stained for pStat3 (green) and Dapi (blue) by immunofluorescence. C. MCF10A cells expressing control (C) or Stat3 shRNA (S3sh) were plated in six-well dishes and cell numbers were determined daily for seven days. Each data point represents the mean value from triplicate wells. [file bcr2725-S2.TIFF]

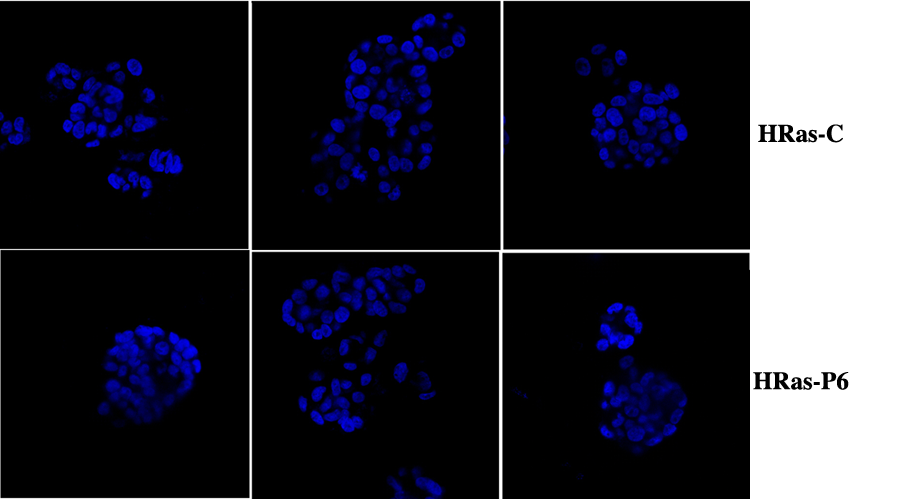

Supplement: Additional file 3 — Supplemental figure S3. Jak inhibition does not alter the morphology of MCF10A-Ras cells in Matrigel. MCF10A-Ras cells were grown on Matrigel and structures were stained for Dapi by immunofluorescence treated with DMSO control (Ras-C) or P6 (a pan-Jak inhibitor) for one week. [file bcr2725-S3.TIFF]
